# A qualitative analysis of the barriers and enablers faced by Australian rural general practitioners in the non-pharmacological management of congestive heart failure in community dwelling patients

**DOI:** 10.1186/s12913-021-07383-3

**Published:** 2022-01-02

**Authors:** Trevor Kwan, Benjamin Chua, David Pires, Olivia Feng, Natalie Edmiston, Jo Longman

**Affiliations:** 1 Western Sydney University, Penrith, New South Wales Australia; 2 University Centre for Rural Health, Lismore, New South Wales Australia; 3 The University of Sydney, Sydney, New South Wales Australia

**Keywords:** General practice, Barriers, Enablers, Non-pharmacological management, Congestive heart failure, Rural Australia

## Abstract

**Background:**

Congestive heart failure (CHF) is a significant health problem in Australia, and disproportionately affects rural Australians. Management of CHF in Australia is heavily centred around the general practitioner (GP). Australian and international literature indicates there is a gap between current and best practice in CHF management. There is little known about the non-pharmacological aspects of management, or CHF management in a rural Australian context. This study aimed to identify what Australian GPs practicing in the Northern Rivers Region of New South Wales, Australia, perceived were the barriers and enablers in the non-pharmacological management of CHF amongst community dwelling patients, to inform healthcare access, resourcing and delivery in Australian rural environments.

**Methods:**

Qualitative study involving a realist thematic analysis of data collected from semi-structured face-to-face interviews.

**Results:**

Fifteen GPs and GP trainees participated. Four interlinked key themes underpinning GPs’ experiences with non-pharmacological management of CHF were interpreted from the interview data: (1) resources, (2) complexity of heart failure, (3) relationships, and (4) patient demographics, priorities and views affect how patients engage with non-pharmacological management of CHF.

**Conclusion:**

Rural Australian GPs face considerable barriers to non-pharmacological management of CHF. The data suggests that increased rural Australian health services and community transportation, multidisciplinary management, and stronger professional networks have the potential to be invaluable enablers of CHF management. Further research exploring non-pharmacological management of CHF in other rural contexts may provide additional insights to better inform rural healthcare access and resourcing.

## Introduction and background

The prevalence of congestive heart failure (CHF) in Australia is estimated to range between 2.1 to 4%, with an incidence of 30,000 newly diagnosed cases every year [1–3]. Internationally, the total number of cases of CHF is thought to exceed 38 million across both urban and rural environments [3]. Global heart failure mortality increases with worsening socioeconomic disadvantage, with the highest levels of mortality in regions such as Africa and India [4]. In Australia, CHF has a higher prevalence amongst certain populations, such as rural communities and Indigenous Australians [1, 5–8]. Like many Indigenous populations internationally, Indigenous Australians experience higher rates of morbidity and mortality in comparison to their non-Indigenous counterparts [9, 10]. Socioecomic inequalities are a potent driving force for these disaparities, along with historical intergenerational trauma, loss of social and cultural capital, and racism [9]. These factors pose unique challenges for treatment which needs to leverage the considerable strengths of family and community amongst this population [11].

CHF is almost always accompanied by one or more chronic comorbidities [5, 12]. It places a high burden on the hospital system, with CHF being the sixth most common cause of potentially preventable hospitalisation (PPH) in Australia [13]. PPHs are defined under the Australian National Healthcare Agreement as unplanned hospitalisations considered to be potentially preventable with timely and adequate outpatient care [13, 14]. Australian Institute of Health and Welfare data indicate that almost half of all PPHs in 2017–18 were attributable to chronic disease, with CHF accounting for 18.2% of this category [13]. One Australian study which assessed admissions with principal discharge diagnoses of CHF found that 63% were actually preventable in the 3 months leading up to admission [14]. Prevention of these admissions through improved care in the community is therefore a priority.

Australia’s healthcare system is underpinned by a government-funded, universal health insurance scheme (Medicare) [15]. Medicare provides heavily subsidised or free access to essential health services for eligible Australians [15]. Services such as allied health, GPs and specialists are typically subsidised, whereas inpatient services at public hospitals are free [15]. Many Australians additionally purchase private health insurance, which covers excess fees, elective procedures, and inpatient services at private hospitals not covered by Medicare [15].

In Australia, diagnosis and management of CHF is heavily centred around general practitioners (GPs) as coordinators of multidisciplinary teams, where access to specialists, allied health providers and diagnostic investigations are made available through referral processes [2, 3]. Timely and accurate diagnosis enables appropriate management decisions and access to services such as cardiac rehabilitation, which for CHF requires a formal diagnosis by echocardiogram [3, 16, 17]. Cardiac rehabilitation is a medically supervised allied health service that delivers non-pharmacological interventions directed towards improving cardiovascular health for patients with cardiac disease [16]. Key responsibilities for GPs in long-term management include monitoring for disease progression, patient education, medication titration and the development and implementation of non-pharmacological management plans [3]. Where available, chronic disease nurses assume many of these same key responsibilities in both primary and secondary health care settings.

Non-pharmacological management encompasses education of patients, their families and carers; support from allied health clinicians; cardiac rehabilitation programs; and a spectrum of behavioural patient self-management strategies including: daily weight monitoring; daily monitoring for signs and symptoms of disease progression (e.g. shortness of breath, orthopnoea or dizziness); sodium and fluid restriction; compression stockings; cessation of smoking; reduction of alcohol consumption; regular exercise; and appropriate and timely help-seeking [3, 18]. Non-pharmacological management of CHF reduces rates of hospitalisation, prolongs the time patients remain out of hospital, reduces mortality, and improves quality of life [3].

Despite Australian guidelines for the management of CHF, there remains a gap between current and best practice CHF management [2, 3, 5, 7, 8, 18, 19]. In the rural Northern Rivers region of New South Wales (NSW), Australia, where this study is set, unpublished data of 65 CHF patients engaged with cardiac rehabilitation services illustrated that 57% sought treatment more than 2 days after worsening symptoms, 29% did not weigh themselves daily, 26% did not restrict their fluid intake, and 11% did not restrict their sodium intake (personal communications to JL).

### Literature review

There is considerable literature on both the barriers and enablers of the management of CHF. In order to understand the existing literature, we applied the Theoretical Domains Framework (TDF) to organise findings about the barriers and enablers faced by GPs in CHF management [20]. The TDF is a theoretically grounded framework for identifying barriers and enablers to behavioural change, such as those recommended in clinical guidelines [21]. Its constituent 14 domains are a distillation of psychological constructs from behaviour change theory [13, 14].

In the Knowledge domain, GPs reported a limited understanding of CHF including classification, progression, prognosis and best-practice management [22–35]. This was complicated by a lack of patient understanding of their diagnosis and a view that CHF can be cured [22, 23, 36].

In the Skills domain, GPs identified dated medical training as a barrier, although additional training was reported to improve care [23, 27, 37]. GPs also reported difficulties with managing CHF patients with multiple comorbidities due to inadequate training [23, 25, 28].

Within the Professional role and identity domain, studies identified an absence of clear professional role definition, absolution from management, and the burden of patient follow-up and monitoring as barriers. The net effect was that many GPs felt acutely presenting patients routinely required specialist referral and hospital admission [23, 28, 34, 35, 37].

In the Beliefs About Capabilities domain, there was an evident lack of confidence amongst GPs in CHF management and communication of diagnosis and prognosis of CHF to their patients [23, 25, 27, 28, 32, 34].

Within the Environmental Context and Resources domain, geography, distance from rural practices, and long referral wait-times were identified as potential barriers [23, 35, 38–40]. This was further complicated by a lack of key resources, including consultation time, community-based rehabilitation facilities, low cost or subsidised treatments, end-of-life care, and an adequately trained workforce [2, 23, 27–30, 35, 38]. Low socioeconomic status (SES) was identified as a barrier to patient engagement with health services and adherence to management, while those with family support demonstrated better self-management [41, 42]. Organisational culture also posed challenges such as compromised communication due to hierarchical boundaries; difficulty communicating with specialists and other clinicians; lack of clear reports and interdisciplinary culture; lack of trust in other healthcare professionals; and difficulty accessing palliation pathways. Additionally, despite being aware of clinical guidelines, GPs often found their recommendations difficult to implement as guidelines were perceived to overlook patient comorbidities and polypharmacy [5, 23, 25, 28, 34, 35, 37]. GPs identified multidisciplinary chronic disease management services and resources as important enablers in improving CHF outcomes [19, 40, 43].

In the Emotions domain, previous research has identified fears pertaining to the initiation, modification, and evaluation of risks versus benefits of management plans; incorrect diagnosis; and loss of patients to other clinicians [23, 25, 27, 32, 34, 35]. Apprehension using clinical terms such as “heart failure” during consultations was also identified [23, 27, 28, 35].

Despite extensive literature on the barriers and enablers of CHF management, much of it focuses explicitly on pharmacological management, with scarce literature available on non-pharmacological management. In addition, while the experiences of both international and Australian GPs who manage patients with CHF in urban environments have been widely represented, the experience of rural GPs remains largely unknown [1, 22–31, 34, 35, 37, 38, 44]. Australian rural GPs are distinct on both an international (Australia versus other developed countries) and domestic (rural versus metropolitan) level, given Australia’s unique geography and healthcare system.

The aim of this study was therefore to identify what Australian rural GPs perceived were the barriers and enablers in the non-pharmacological management of CHF amongst community dwelling patients, in order to better inform healthcare access, organisation, resourcing, and delivery in Australian rural environments.

Barriers are defined in this study as a person, object or circumstances (experiences, values, perspectives or knowledge) that prevents or makes patient management difficult, whereas an enabler facilitates management.

## Methods

### Participants and recruitment

A purposive sample of potential participants from general practices and local networks was approached with the aim of maximising variation in participants’ training and practice experience, as well as the type of practice and demographic profile of their patients.

GP and GP trainees who had experience managing at least one community dwelling patient with CHF at any point in their training or practice as GPs were included. Following initial recruitment, further recruitment was aided by adoption of a snowball sampling approach [45].

### Study location

The Northern Rivers is a rural coastal area of northern NSW, Australia. The region has a population just over 250,000 people and higher proportions of Indigenous and geriatric (age > 65 years) populations compared to the rest of the state [46]. It is overrepresented by areas of socioeconomic advantage and areas of disadvantage compared to NSW averages [46].

### Data collection

Data were collected through audio-recorded, semi-structured face-to-face interviews with participating GPs and up to two interviewers. This approach facilitated the collection of open-ended data, in the form of GPs’ thoughts, feelings and experiences, and aimed to provide a pragmatic and detailed account of their management of CHF. Participating GPs were provided with information regarding the study and informed written consent was obtained from all participants. Interviewers (TK, BC, DP and OF) were fourth year medical students on 12-month rural placements in the Northern Rivers, and received training and support in qualitative data collection from an experienced qualitative researcher (JL). An interview schedule based on the research question and findings from the literature review was developed and piloted with minor amendments subsequently made in structure and content prior to use in data collection.

### Data analysis

To ensure methodological reliability, a realist thematic analysis was conducted in accordance with the steps outlined by Braun and Clarke [47]. To immerse and familiarise researchers with the data, the audio-recordings were transcribed verbatim by the researcher who had conducted the interview, and de-identified before being proofread by another researcher to check for accuracy of transcription.

A rudimentary code index was developed from existing literature by BC [3, 28, 38] and a data-rich transcript deductively coded to explore fit. To refine the code index, the five most data-rich transcripts were then deductively coded by TK and OF using the rudimentary index, and additional inductively-derived codes were added to the index following extensive discussion across the research team. A coding comparison query run on a data-rich transcript, independently coded by TK and OF, was used to resolve discrepancies in the application of the code index, leading to its finalisation. This final code index was used by TK and OF to then code all transcripts.

Codes and relevant extracts were collated into categories and then eventually into candidate themes, aided by a thematic map to visually represent relationships between codes. Candidate themes were reviewed by first assessing the collated extracts. This was followed by a review of the thematic map and individual themes to ensure they accurately reflected the entire data set and were relevant to the study aim. To conclude, extensive discussion helped refine each theme and to develop the overall account from the thematic analysis.

## Results

A total of 17 GPs were invited to participate in the study. Two declined to participate due to time constraints. Interviews lasting an average of 28 min (ranging 16–45 min) were conducted with the remaining 15 participants. The recruited sample displayed significant heterogeneity, with variation in training and practice experiences, duration of practice and exposure to patients with CHF (Table 1).

**Table 1:** Participant Information

GP number	GP or GP trainee	Years of practice	General demographic profile of all patients seen	CHF patient load^a^	Profile of CHF patients
1	GP	Newly registered	∙ Low socioeconomic status (SES) ∙ Retirees ∙ Young families	Medium	–
2	GP	4	∙ Farming families ∙ Retirees ∙ Young families	High	∙ Elderly (> 65 years old) ∙ Male ∙ Farmers
3	GP	20	∙ Young families ∙ Elderly (> 65 years old) ∙ Indigenous	Medium	∙ Multi-morbidity
4	GP	6	∙ Indigenous	Medium	–
5	Trainee	0.5	∙ Women ∙ Children ∙ Elderly	Low	∙ Elderly (> 65 years old) ∙ Young ∙ Pre-existing cardiac conditions
6	GP	45	∙ Indigenous ∙ Young families	Medium	∙ Elderly (> 65 years old)
7	GP	30	∙ Indigenous	Medium	∙ Patients with risk factors for cardiac disease (smoking, obese, sedentary)
8	GP	13	∙ Lower SES ∙ Indigenous ∙ Substance use disorders	Low	∙ Low SES ∙ Indigenous ∙ Substance use disorders ∙ Young ∙ Pre-existing cardiac conditions
9	Trainee	1	∙ Indigenous	Low	∙ Indigenous
10	GP	20	∙ Indigenous	High	∙ Indigenous ∙ Multi-morbidity ∙ Low SES
11	GP	30	∙ Young ∙ Elderly	Medium	–
12	GP	14	∙ Indigenous ∙ Tourists ∙ High SES	Medium	∙ Elderly (> 65 years old)
13	GP	6	∙ Varied	N/A	∙ Elderly (> 65 years old)
14	GP	9	∙ Tourists ∙ High SES	Medium	∙ Elderly (> 65 years old)
15	GP	15	∙ Young	High	∙ Elderly (> 65 years old) ∙ Pre-existing cardiac conditions

^**a**^High: managing ≥5 CHF patients total, ≥1 seen daily; Medium: managing ≥5 CHF patients total, < 1 seen daily; Low: managing < 5 CHF patients total

### Main findings

Four key overarching and interlinked themes were interpreted (Table 2).

**Table 2:** Themes and subthemes

Theme	Subtheme
Resources	Distance to services
	Inadequate consultation time and remuneration
	Inadequate service availability
Complexity of heart failure	Patients’ understanding of disease
	Comorbidities
	Coordinated multidisciplinary care
Relationships	Relationships between GPs and cardiologists
	Relationships between GPs and their patients
Patient demographics, priorities and views	Socio-economic disadvantage and patients’ personal priorities
	Patients’ family-level barriers
	Patients’ views

### Theme 1 - resources

#### Distance to services

Distance to rural allied health services was consistently described as a barrier to patient engagement. The relative scarcity of these services and their geographical dispersion often meant that many patients had to travel significant distances to attend appointments. Additionally, many GPs lamented that these issues were frequently exacerbated by the region’s minimal community transport options. Patient comorbidities and family commitments only further compounded the difficulties with rural and regional travel.

#### Inadequate consultation time and remuneration

Many GPs described how inadequate consultation time and remuneration impacted on their ability to provide comprehensive care.

“We can't necessarily spend the time …we’ve actually got to generate an income in a relatively insufficient time frame …it frustrates me … I think a lot of that care should be done in general practice… you just feel a bit pressured with time.”

- GP 008

One GP explained why a majority of their colleagues in the Northern Rivers elected to charge their patients beyond the rate of Medicare subsidies, rather than reduce their fees such that consultations were entirely compensated by Medicare subsidies (known as bulk billing), as choosing to do so would lead to an unsustainable workload.

“Very few doctors bulk bill or advertise that they bulk bill. I think a lot of them actually do but they don't advertise they do so they're not gonna sort of end up with a huge, they basically manage their work load by access blocking.”

- GP 008

Significant patient loads minimised the time that GPs could spend developing ongoing therapeutic relationships with their patients and limited the opportunities for patient education.

Conversely, GPs noted that government-funded initiatives promoting cardiac screening and chronic disease management were essential enablers, as GPs could be remunerated for screening and management of CHF. This was described by many GPs to be a strong incentive for providing comprehensive and holistic care for patients with complex conditions like CHF.

#### Inadequate service availability

GPs described difficulties with CHF patients accessing echocardiograms for diagnostic purposes, due to barriers such as high cost, unavailability, and protracted wait times. These same barriers were raised when discussing access to cardiologists in the Northern Rivers. Together, these barriers delayed accurate diagnosis and subsequently encumbered timely management decisions. Additionally, without a formal diagnosis, many patients were ineligible for services such as cardiac rehabilitation.

### Theme 2 – complexity of heart failure

#### Patients’ understanding of disease

GPs reported that the inherent complexity of CHF was a barrier to delivering patient education and improving patient understanding. It routinely translated to difficulties adequately conveying and contextualising the importance of non-pharmacological management and thus was seen to potentiate patient non-adherence to suggested non-pharmacological self-management strategies.

“Most of the problems tend to be around the education. Getting the patients onboard, understanding what their early warning signs are, getting them to understand that they're in control….to keep their weight and fluid levels down, and then being able to monitor for other warning signs. Whether it be irregular heartbeats, tolerance, distance, ability to lie flat, how many pillows they're using.”

- GP 004

One GP postulated that lower levels of health literacy in the area may be contributing to their patients’ limited understanding of CHF.

#### Comorbidities

Another key barrier to CHF management identified by several GPs was comorbidity. Pre-existing comorbidities and their complications were reported to reduce a patient’s tolerance for further interventions such as fluid restrictions, compression stockings and exercise, and hence GPs described difficulties tailoring their management for individual patients.

“Another sort of main challenge is just that these people obviously often are quite a bit older and they’ve had these various different sorts of chronic conditions quite some time and ultimately becomes that sort of balance… what are their goals, what are their lives… trying to kind of tailor the treatment appropriately.”

- GP 003

#### Coordinated multidisciplinary care

Multidisciplinary management of CHF was consistently viewed as a critical enabler. When appropriately encapsulated as a care plan, GPs noted improved patient disease understanding, engagement with health services, and adherence to prescribed non-pharmacological management.

“[It’s] pretty useful where you have ... not just a cardiologist but you've got allied health and it's kind of like a multidisciplinary approach, …that can be helpful for patients in terms of being educated about the disease …, getting different advice from different perspectives and I think that can be quite encouraging for patients as well.”

- GP 005

Cardiac rehabilitation was stressed to be particularly pivotal in improving outcomes in both early and ongoing management. Moreover, chronic disease nurses were commonly described to play an essential role in the effective planning and coordination of continuous care for CHF patients.

“Having a cardiac nurse in primary care whose job was to manage that cohort of people in primary care ..., is really beneficial. I just think having someone to manage their complex care from a health background, whether it be a hospital or in primary care, is undoubtedly very helpful.”

- GP 008

They were also noted to be invaluable when reinforcing education regarding non-pharmacological management and monitoring for disease progression.

“It just comes back to having nurses who are always around… who can just run a really good preventive health service and work together with the GPs... if they know that the nurse is going to give them the same information the doctor's going to give them and that service is always there and they can come in when things aren't going well to plan or... when we're trying to educate them, I think it's so much more beneficial”

- GP 009

Some GPs anticipated that provision of more co-located multidisciplinary healthcare providers within culturally sensitive systems such as Aboriginal Medical Services (AMSs) would foster more positive therapeutic relationships and facilitate the ongoing delivery of non-pharmacological CHF management to Indigenous communities.

“Having [allied health practitioners] on site especially in an Aboriginal medical service would just be so helpful. We do lots of little other things so, to explore that a bit more would be so beneficial and keeping it in-house I think in an [Indigenous] population would make them I guess, comes back to that relationship with your care providers, and I think that would be really really helpful for them.”

- GP 009

On the other hand, fragmented care due to poor communication between providers at different levels of healthcare was commonly reported. Many GPs emphasised the discordance between primary and tertiary health care systems, particularly regarding delayed discharge summaries which disrupted continuity of care. This issue was interpreted by one GP to be due to the sole focus on acute care over promoting primary prevention in tertiary settings.

“It's a bit of a pet peeve how bad communication can be sometimes and even how hard it can sometimes be for some specialists... to be able to get them on the phone to be like ‘I'm having troubles with managing this person you see’... discharge summaries come back late... patient care then and there is probably top priority. I just think if we all communicated a lot better and the service is linked in a bit better... I think that would really help people manage chronic disease better.”

- GP 003

### Theme 3 – relationships

#### Relationships between GPs and cardiologists

Established professional relationships between GPs and cardiologists were described to benefit patient care coordination and enable non-pharmacological management planning for patients with CHF. GPs attributed these closer connections to the smaller number of cardiologists within the area.

"Having worked locally in the local base hospitals, I've worked with all the local cardiologists and so I know them well... a quick 2-minute conversation can save three months and an avoidable admission to hospital. So that's really valuable and that is a great enabler."

- GP 001

However, ambiguity in relationships between GPs and cardiologists was described as a barrier to CHF management, as some GPs felt it resulted in a lack of clear professional role in their patient’s ongoing management.

#### Relationships between GPs and their patients

Trust, rapport and effective communication with patients were perceived as significant therapeutic enablers which fostered continuity of care, patient understanding and long-term self-management. Some GPs, particularly in AMSs, explained how positive relationships with a patient’s family members often improved patient adherence as it facilitated patient and family-centred decision making.

“It’s about having conversations and really trying to understand... the relationships I think are key to that, they need to know you, you need to know them, and then you’re more likely to get a bit of traction.”

- GP 003

On the other hand, one GP expressed how inadequately explaining the rationale for non-pharmacological management often meant that patients would struggle to identify its personal relevance and therefore tended to be less adherent.

"Clinicians don't explain the reasons for their care well enough... The importance of certain things is not demonstrated or discussed in the right context with the patient to identify why it's important to them personally."

- GP 008

Many GPs noted that patient-centred care and goal-oriented management were important principles to consider when implementing non-pharmacological interventions. One GP described how collaboratively setting measurable and achievable goals with their patients, based on compromise, individual circumstances and shared accountability encouraged and motivated their patients to improve adherence with non-pharmacological self-management.

“My little template, is “okay between now and when I see you again, what are our goals? And I’m going to hold them to you, and they can be one or two”; and if it was related to their heart disease, it’ll be like “okay, well, this is what you need to fluid restrict to this much and how we’re going to do that. That’s our goal” and just break it down in chunks, then we revisit it in two to three months time, or earlier if I need to”

- GP 008

### Theme 4 – patient demographics, priorities and views

#### Socio-economic disadvantage and patients’ personal priorities

Many GPs identified socio-economic disadvantage and personal priorities as significant barriers to patient engagement with self-management of CHF. GPs commonly described how patients experiencing economic disadvantage viewed their health as less important than other immediate personal priorities, such as ensuring they had food and housing. Many of these patients struggled to adhere to dietary restrictions as an appropriate healthy diet was comparatively more costly than cheap processed food options with high sodium contents.

This economic disadvantage was accentuated by the high proportion of privately practicing allied health services within the Northern Rivers. Many GPs described their patients’ inability to pay an upfront service fee as a barrier to receiving allied health input in the development and ongoing implementation of non-pharmacological management plans. Financial barriers to accessing specialist services were also reported. However, several GPs remarked that their patients’ experiences negotiating service fees with cardiologists in the Northern Rivers were generally positive, as cardiologists were often willing to bulk bill vulnerable populations. AMSs were described as assisting Indigenous patients in overcoming financial barriers by delivering subsidised, coordinated care.

“The AMS [patients] are quite lucky because if they have chronic health conditions … you can develop a management plan … and [government subsidies] will pay for a private visit to a cardiologist.”

- GP 008

#### Patients’ family-level barriers

The influence of family and carers was regarded by most GPs to be a significant barrier to their patients’ self-management of CHF. For instance, due to a limited understanding of CHF, some family and carers offered beverages to fluid-restricted patients as they were unaware that this would exacerbate their condition.

Additionally, many GPs observed that the culture of the Northern Rivers was unique in that there was a prevalent distrust of mainstream Western medicine. Many patients instead favoured alternative medicines and practices, such as herbal medicines, homeopathy and kinesiology. One GP recounted how their experiences with family and carers that preferred alternative medicine was challenging as their beliefs often complicated medical decision making, particularly for their older patients where their families and carers had power of attorney.

Some GPs noted a barrier to management of CHF was GPs’ lack of cultural understanding around shared decision-making in Indigenous communities, which are typically tight-knit and operate as collective family units.

There’s a distinct inability to understand the way people make decisions in the [Indigenous] community and the need for allowing whoever needs to be in the room to make the decision and having that process…. [Indigenous people] need to understand why it’s important, how it impacts, who it impacts, you know, they need to ask the important people in their life about whether that’s a good thing for them or not.

- GP 008

#### Patients’ views

GPs identified several key patient views to be barriers to effective management of CHF as these views dampened engagement with services. One GP highlighted a poor sense of agency amongst their Indigenous patients, attributed to chronic disempowerment and intergenerational trauma, affecting their perceived ability to implement meaningful lifestyle changes, such as exercise regimens or diet plans.

“There’s a different attitude to empowerment, to being able to be a master of your health destiny... a lot of the people that we see here as patients historically have been disempowered and chronic racism and ...socio-political history. They are not empowered to feel like they’re going to be able to be masters of [lifestyle changes] in their life.”

- GP 007

Another view was the expectation that management came in the form of a ‘magic pill’, in that patients had preconceived expectations of an instant cure-all solution to manage their CHF. Several GPs described that this expectation detracted from their patients’ desire to implement non-pharmacological management focused on long-term incremental gains, as they quickly became disheartened when tangible gains were not immediately evident. Furthermore, this led to patients dismissing non-pharmacological interventions such as exercise, fluid restriction or dietary changes as they believed a far easier ‘Quick Fix’ alternative existed.

“Poor patient adherence to non-pharmacological management, like I think it’s the key stone of everything and I think this is why we have so much or not all chronic diseases attributed to this but a lot of it is the fact that we just don’t look after ourselves. We’re kind of waiting for the magic pill to fix everything and it’s not there and then patients get distressed because they’re not seeing results and yeah, you know, they take the easiest path to their treatment.”

- GP 009

Finally, many GPs described their patients’ views towards their lifestyle were often cemented by long-standing patterns of behaviour. As a result, lifestyle modifications for older patients were often regarded as inherently difficult.

“If you’re 70 or 75 …, then it’s hard to change the way you live because you’ve lived like that for 50 years.”

- GP 012

## Discussion

This study provides insight into rural GPs’ experiences and perceptions of the barriers and enablers of non-pharmacological management of CHF. Systemic barriers to diagnosis and management included inadequate systemic resource availability, disease complexity and poor communication between the primary and tertiary levels of healthcare. However, government funded programs and strong relationships between GPs and patients, cardiologists and allied health staff were recognised as significant enablers aiding management. Patient-level barriers, such as low SES, personal priorities, familial influence, and healthcare views, all negatively impacted self-management.

Distances to services and limited transportation options have been consistently described as systemic barriers to rural service access which directly reduce patient engagement with healthcare services [23, 35, 38–40]. This was a sentiment shared by many GPs in this study, indicating there is an ongoing need for increased rural health services and community transportation. Additional consideration for rural community transport may be required for patients with comorbidities or significant family commitments as their access to health services was reported to be particularly impaired. Telecommunication technologies in areas with few health services may supplement existing services in facilitating symptom and treatment outcome monitoring, and enhancing patient motivation and education, despite physical barriers to rural service access [48].

In this study, inadequate consultation length was often attributed to significant patient loads and inadequate remuneration. Previous research has shown a strong correlation between inadequate GP consultation length and shortcomings in CHF management and patient education [23, 28, 38, 49]. While financial pressures were described to be partially alleviated by government incentives that promoted cardiac screening and chronic disease management, continually overwhelming patient loads suggests that rural areas remain chronically underresourced and underserviced.

Potentially exacerbated by inadequate consultation length, GPs also described their struggle to adequately explain the complex pathophysiology of CHF to their patients. One GP drew attention to how this was compounded by low patient health literacy. Improved patient understanding was frequently described to have benefitted from multidisciplinary education, spearheaded by chronic and cardiac disease nurses. It may therefore be beneficial for more GPs to entrust the professional roles and responsibilities of patient education with subspecialised community nurses. These findings also signal the need for further skills training for GPs geared towards patient education, which will not only improve patient education but also strengthen GPs’ professional confidence in their capabilities.

Beyond patient education, GPs in this study highlighted the importance of cardiac rehabilitation and chronic care nurses in the implementation and coordination of care, which is pertinent as multidisciplinary team arrangements are often complicated by issues of accessibility and fragmentation of care between providers [23, 37]. Additionally, AMSs were described to be particularly valuable for Indigenous patients in providing coordinated, subsidised, and culturally appropriate care for Indigenous communities.

Poor communication between different levels of healthcare has been documented to be a key barrier to effective CHF management and corroborated by the findings of this study, illustrating the need for more effective communication channels [36, 49]. A recent regional Australian study exploring discharge of stroke patients found that key factors contributing to delayed discharge summaries included workload pressures; pressures to discharge patients quickly and at short notice due to bed demands; and inadequate information available to the persons responsible, often the most junior member of the team [50]. Whilst this paper addressed stroke patients in a different part of regional Australia, these key themes are consistent with the local experience (personal communications to TK). GPs in this study highlighted that their positive professional relationships with members of their smaller rural networks were enablers in CHF management, highlighting the importance of fostering rural networks.

Family support has been previously identified as an enabler for self-management of CHF [42]. Contrastingly, GPs in this study often described familial influences as counterproductive to self-management. This notion was particularly unique amongst Indigenous patients, where the importance of family units was not accommodated for in individual lifestyle recommendations. These findings suggest that family education should be considered more routinely, as it was seen to improve family support towards individual lifestyle modifications and adherence to prescribed non-pharmacological management. Initiatives increasing uptake and engagement with AMSs may have utility in navigating the community contexts in which Indigenous patients live, which are often underpinned by a compromised sense of agency and chronic disempowerment.

Other barriers to self-management not addressed by participants in this study, but evident in literature, include anxiety and depressive disorders. They are recognised to be frequent in cardiac patients but are often underrecognised, and are seen to interfere with the adoption of cardiac self-care and risk-reducing behaviours [51].

Patients’ distrust of health services and mainstream Western medicine, and the opposing view that management could comprise of a ‘magic pill’, were both perceived to be barriers to CHF management and are novel findings of this study. Distrust of mainsteam Western medicine is recognised to be prevalent in the Northern Rivers [52]. GPs in this study also recognised that lifestyle modifications for their older patients were inherently difficult due to long term, established patterns of behaviour, a view not yet described in existing literature. One study suggests that older patients may have a better understanding of their chronic conditions although this study has found nothing to suggest that this has translated to improved adherence with non-pharamcological management [53].

### Strengths and limitations

The snowball sampling approach used in this study has the potential to limit the participant sample to those with similar perceptions, experiences, and biases regarding CHF management [54, 55]. Furthermore, two GPs declined to participate in the study. These aspects of recruitment may lead to certain GPs’ voices not being captured in the data. However, there was considerable diversity within the sample, with significant variation in training and practice experience, as well as the type of practice and demographic profile of patients. All participants serviced areas within the Northern Rivers, which may limit the applicability of this study’s findings to other Australian or international rural or metropolitan communities.

## Conclusion

Rural Australian GPs face considerable barriers to non-pharmacological management of CHF at systems, clinician and patient levels. The data portrays a significant need for improved rural Australian health care resourcing and accessibility; re-organisation of the rural health workforce; and increased uptake and development of available rural health services in response to the significant health burden of CHF which disproportionately affects rural Australians. Decisions on rural healthcare resourcing may be better guided by further research exploring non-pharmacological management of CHF in other rural contexts.

## Data Availability

The datasets used and/or analysed during the current study are not publicly available as participants have consented for their data to be available only to researchers of this study. They may be made available from the corresponding author upon reasonable request.

## Abbreviations

CHF: Congestive heart failure
GP: General practitioner
NSW: New South Wales
AMS: Aboriginal medical service
